# Supplementary material for: Brain structures and activity during a working memory task associated with internet addiction tendency in young adults: A large sample study
Source: PLoS One. 2021 Nov 15;16(11):e0259259. doi: 10.1371/journal.pone.0259259 (PMC8592411; doi:10.1371/journal.pone.0259259)
Supplement: S1 Table — Abbreviations: FDR, false discovery rate; unc, uncorrected; L, left; R, right; mPFC, medial prefrontal cortex; PCC, posterior cingulate cortex. (DOCX) [file pone.0259259.s001.docx]

**S1 Table.** Supplemental ROI analyses of associations between activity in key nodes of the DMN and IAT scores

| Lobe (L/R) | 0-back | | | 2-back | | |
| --- | --- | --- | --- | --- | --- | --- |
|  | Partial correlation coefficients | *P* (unc) | *P*  (FDR) | Partial correlation coefficients | *P* (unc) | *P*  (FDR) |
| mPFC | 0.062 | 0.035 | 0.060 | 0.126 | 0.0002 | 0.0002 |
| PCC/precuneus | 0.023 | 0.434 | 0.474 | 0.067 | 0.024 | 0.060 |
| Left hippocampus | 0.041 | 0.165 | 0.220 | 0.090 | 0.002 | 0.014 |
| Right hippocampus | 0.032 | 0.272 | 0.326 | 0.079 | 0.007 | 0.028 |
| Left temporoparietal junction | 0.063 | 0.032 | 0.060 | 0.052 | 0.078 | 0.117 |
| Right temporoparietal junction | 0.065 | 0.028 | 0.060 | -0.01 | 0.730 | 0.730 |
