# Supplementary material for: Brain structures and activity during a working memory task associated with internet addiction tendency in young adults: A large sample study
Source: PLoS One. 2021 Nov 15;16(11):e0259259. doi: 10.1371/journal.pone.0259259 (PMC8592411; doi:10.1371/journal.pone.0259259)
Supplement: S1 Abbreviations — (DOCX) [file pone.0259259.s002.docx]

**Abbreviations**

3 Tesla (3T)

anterior cingulate cortex (ACC)

centimeter (cm)

central executive (CE)

default-mode network (DMN)

diffusion tensor imaging (DTI)

dorsolateral prefrontal cortex (DLPFC)

echo planar imaging (EPI)

echo time (TE)

false discovery rate (FDR)

field-of-view (FOV)

functional magnetic resonance imaging (fMRI)

gambling disorder (GD)

gamma aminobutyric acid (GABA)

gray matter volume (GMV)

hyperactivity and attention disorder (ADHD)

internet addiction (IA)

internet addiction disorder (IAD)

internet addiction tendency (IAT)

magnetic resonance imaging (MRI)

mean diffusivity (MD)

medial prefrontal cortex (mPFC)

millimeter (mm)

millisecond (ms)

Montreal Neurological Institute (MNI)

orbitofrontal cortex (OFC

post-central gyrus (postCG)

posterior cingulate cortex (PCC)

pre-supplementary motor area (pre-SMA)

region of interest (ROI)

regional gray matter volumes (rGMVs)

regional white matter volumes (rWMVs)

repetition time (TR)

right inferior frontal gyrus (rIFG)

right supramarginal gyrus (rSMG)

rostral anterior cingulate cortex (rACC)

sensitivity encoding (SENSE)

Statistical Parametric Mapping (SPM)

substance use disorders (SUD)

subthalamic nucleus (STN)

supplementary motor area (SMA)

task-induced deactivation (TID)

threshold-free cluster enhancement (TFCE)

voxel-based morphometry (VBM)

white matter volume (WMV)

working memory (WM)
