# Supplementary material for: Brain structures and activity during a working memory task associated with internet addiction tendency in young adults: A large sample study
Source: PLoS One. 2021 Nov 15;16(11):e0259259. doi: 10.1371/journal.pone.0259259 (PMC8592411; doi:10.1371/journal.pone.0259259)
Supplement: Attachment — Submitted filename: Response_to_Reviewer.docx [file pone.0259259.s006.docx]

**Response to Reviewers**

**Manuscript ID:** PONE-D-21-09795
**Manuscript title:** Brain Structures and Activity During a Working Memory Task Associated with Internet Addiction Tendency in Young Adults: A Large Sample Study
**Journal**: PLOS ONE

Dear editor and reviewers,

We sincerely thank the editor and reviewers for constructive and valuable comments, which were of great help in revising the manuscript. Accordingly, the revised manuscript has been systematically improved with new information and additional interpretations. Our responses to the editor comments (**AR_e_**), Journal Requirements (**ARjr**) and referee’s comments (**AR_r_**)are given below. Also, green text indicating changes has been added to the revised manuscript.

**Editor comments**

**EC 1**. "Do not include funding sources in the Acknowledgments or anywhere else in the manuscript file. Funding information should only be entered in the financial disclosure section of the submission system."

**AR_e_ 1**. Thank you for your comment. We deleted the funding sources information in Acknowledgments and in the manuscript file. Funding information just entered in the financial disclosure section of the submission system.

**Reviewer comment**

**RC 1**. Compared to the first draft, important changes have been made in the different sections of the paper. You tried to address the whole points raised by me.

As per the journal requirement “Acknowledgment” session is mandatory. So, try to include it after the conclusion session, and acknowledge people who were contributing for the development of this article.

**AR_r_ 1.** Many thanks for your attention. We added “Acknowledgment” session in the end of the document as below:

**Acknowledgment**

We respectfully thank Yuki Yamada for operating the MRI scanner and Haruka Nouchi for acting as an examiner for psychological tests. We also thank study participants, the other examiners of psychological tests, and all of our colleagues at the Institute of Development, Aging and Cancer, Tohoku University, for their support.

**Journal Requirements**

**JR 1**: Please review your reference list to ensure that it is complete and correct. If you have cited papers that have been retracted, please include the rationale for doing so in the manuscript text, or remove these references and replace them with relevant current references. Any changes to the reference list should be mentioned in the rebuttal letter that accompanies your revised manuscript. If you need to cite a retracted article, indicate the article’s retracted status in the References list and also include a citation and full reference for the retraction notice.

**ARjr**: We reviewed our references carefully and have cited papers that have been retracted.
