# Supplementary material for: Brain structures and activity during a working memory task associated with internet addiction tendency in young adults: A large sample study
Source: PLoS One. 2021 Nov 15;16(11):e0259259. doi: 10.1371/journal.pone.0259259 (PMC8592411; doi:10.1371/journal.pone.0259259)
Supplement: Attachment — Submitted filename: Reviewer Comment @ Plos One.docx [file pone.0259259.s003.docx]

I thank the authors and the section editor for bringing this work to my attention.

It is an interesting proposal “Brain Structures and Activity During a Working Memory Task Associated with Internet Addiction Tendency in Young Adults: A Large Sample Study”. but some clarification is needed:

1. Page 1, 2, 3, 4, 6, 7, 11 and 13; WM, fMRI, GABA, EPI, UK, TFCE, ACC and PCC were not clear. Write them in their long form for their first appearance. Please try to include them in the abbreviation session.
2. Page 4: Why do you prefer to use the right handed participants?
3. Page 5: The IAT scale minimum and maximum scores are 20 and 100, with higher scores reflecting a greater tendency toward internet addiction. The Japanese version of this scale has demonstrated high reliability and validity. What does a higher score of internet addiction test mean? It should be specified clearly based on their degree of severity. As you stated your study participants were students from Tohoku University in Japan and you might have international students out of Japan with different culture, religion and so on. So, have you done a validity test for your study?
4. Page 8: The interaction between sex and the score of Young’s IAT scale (contrasts of [the score of the Young IAT scale for males, the effect of the score of the Young IAT scale for females] were [−1 1] or [1 –1]) were assessed using t-contrasts. Correction for multiple comparisons was performed using the same method used in the whole-brain multiple regression analyses. There is repetition of words as highlighted above. So, remove one of them.
5. Page 9 and Page 11: For this comparison, we conducted multiple regression analyses in which all dependent and independent variables of the main analyses remained the same, except that Young’s IAT score was replaced by the dichotomized value (Young’s IAT scale ≥50 = 1, Young’s IAT scale < 0).What does it mean?
