# Supplementary material for: Brain structures and activity during a working memory task associated with internet addiction tendency in young adults: A large sample study
Source: PLoS One. 2021 Nov 15;16(11):e0259259. doi: 10.1371/journal.pone.0259259 (PMC8592411; doi:10.1371/journal.pone.0259259)
Supplement: Attachment — Submitted filename: Reviewer Comment @ Plos One - 2014.docx [file pone.0259259.s005.docx]

Compared to the first draft, important changes have been made in the different sections of the paper. You tried to address the whole points raised by me.

1. As per the journal requirement “Acknowledgment” session is mandatory. So, try to include it after the conclusion session, and acknowledge people who were contributing for the development of this article.
